# Maternal fish consumption during pregnancy and smoking behavioural patterns

**DOI:** 10.1017/S0007114517003592

**Published:** 2018-03-28

**Authors:** Rachel V. Gow, Jon Heron, Joseph R. Hibbeln, John M. Davis, John Paul SanGiovanni

**Affiliations:** 1 Section on Nutritional Neurosciences, Laboratory of Membrane Biochemistry and Biophysics, National Institute on Alcohol Abuse and Alcoholism, Rockville, MD 20852, USA; 2 School of Social and Community Medicine, University of Bristol, Bristol BS8 1TH, UK; 3 University of Illinois at Chicago, Chicago, IL 60607, USA; 4 Georgetown University School of Medicine, Washington, DC 20057, USA

**Keywords:** Avon Longitudinal Study of Parents and Children, *n*-3 Fatty acids, Smoking, Pregnancy, Addiction, Fish

## Abstract

*n*-3 Highly unsaturated fatty acids (HUFA), are essential components of neuronal membranes and mediate a range of complex bioactive properties including gene expression, myelination, cell-signalling and dopaminergic function. Deficits in *n*-3 HUFA have been linked to increased risks for addictive disorders, thus we posited that lower fish consumption would be associated with greater risks for perinatal smoking among 9640 mothers enroled in the Avon Longitudinal Study of Parents and Children. We used univariable and multivariable regression models to examine relationships between self-reported prenatal dietary intakes of *n*-3 HUFA-rich foods (fish and shellfish) and maternal smoking; outcomes included cessation and the number of cigarettes smoked per d. Both before and during pregnancy, there was consistent evidence (*P*<0·001) of protective fish intake–smoking associations; relative to mothers reporting no fish consumption, those who reported some fish consumption (<340 g/week) and high fish consumption (340 g+/week) at 32 weeks of gestation showed lower likelihoods of smoking (adjusted *P* values <0·001). Respective OR for these relationships were 0·87 (95% CI 0·77, 0·97) and 0·73 (95% CI 0·61, 0·86). Although the prevalence of smoking diminished, from a high of 31·6% (pre-pregnancy) to a low of 18·7% (second trimester), the magnitude of fish intake–smoking associations remained stable following adjustment for confounders. These observations suggest that greater fish or *n*-3 HUFA consumption should be evaluated as an intervention to reduce or prevent smoking in randomised clinical trials.

Maternal smoking during pregnancy is a common and well recognised risk factor for a range of adverse health and developmental outcomes and of significant interest to public health policies and practices^(^ ^1^ ^)^, including alterations in genetically programmed brain development during fetal life^(^ ^2^ ^)^. Smoking rates differ according to maternal age and education/profession. The Office of National Statistics UK, estimated that up to 23 % of women of reproductive age and approximately one in ten babies were born to women who smoked in pregnancy for 2015^(^ ^3^ ^)^. The Avon Longitudinal Study of Parents and Children (ALSPAC) reported that women who smoked during the first trimester of pregnancy were 55 % less likely than their non-smoking peers to breastfeed and 40 % less likely to participate in employment, education or training-based opportunities^(^ ^4^ ^)^. Mothers who smoked during the first trimester were also 50 % more likely than their non-smoking peers to be depressed at 8 weeks postpartum and also had an 1·3-fold higher likelihood of feeling poorly attached to, or hostile toward, their children^(^ ^4^ ^)^.

Tobacco smoke contains more than 500 compounds with putative neurotoxic capacities^(^ ^2^ ^)^. Children exposed to cigarette smoke *in utero* are more likely than their peers to be born at <37 weeks of gestation^(^ ^5^ ^)^, low birth weight, that is, <2·5 kg^(^ ^6^ ^–^ ^8^ ^)^, as still births^(^ ^9^ ^)^, and with congenital malformations^(^ ^10^ ^)^. Adverse perinatal smoking-associated outcomes include alterations in maternal and child brain structure and function which in turn adversely impact behaviour; infants born to mothers who smoke during pregnancy present with smaller head circumferences^(^ ^11^ ^)^, structural alterations in the amygdala^(^ ^12^ ^)^, and volumetric reductions in cortical grey matter, the corpus callosum and frontal, temporal and parietal lobes^(^ ^13^ ^)^. Exposure to tobacco smoke *in utero* is also linked to increased risks of hyperactive-inattentive behaviour^(^ ^14^ ^)^, and a 3-fold increased likelihood of being diagnosed with attention deficit hyperactivity disorder^(^ ^15^ ^–^ ^18^ ^)^. Maternal smoking may impact neurodevelopment via several mechanisms including placenta insufficiency, reductions in blood flow and O_2_ deprivation in the brain^(^ ^19^ ^)^ alternations in fetal brain gene expression^(^ ^20^ ^)^, altered nicotinic receptors^(^ ^21^ ^)^ and persistent alterations in neurotransmitter activity and turnover^(^ ^22^ ^–^ ^24^ ^)^ including an impaired dopaminergic system. Smoking during pregnancy is likely to decrease the amount of blood sent to the fetus and hinder the supply of both nutrition and O_2_ resulting directly on the brain of the fetus via tar and carbon monoxide exposure^(^ ^25^ ^,^ ^26^ ^)^. It is now known that nicotine crosses the placenta, enters the fetal circulation and accrues in the fetal compartments from as early as 7 weeks of gestation^(^ ^2^ ^)^. Identification of novel agents that are safe and may reduce the burden of smoking is useful.

*n*-3 Highly unsaturated fatty acids (HUFA) are biophysically and biochemically essential components of neuronal membranes that are critical for healthy brain development and optimal neurological function^(^ ^27^ ^)^. Zaparoli & Galduroz^(^ ^26^ ^)^ have posited that *n*-3 HUFA, including DHA and EPA, may also have the potential to positively alter smoking behaviours, due to their role in supporting dopamine mediated reward function. One double-blind, randomised, placebo-controlled pilot study reported significant reductions in the number of cigarettes smoked and in tobacco cravings following supplementation with *n*-3 compared with placebo^(^ ^25^ ^)^. A second report found in a cross-sectional observation study lower levels on *n*-3 HUFA among smokers compared with controls and, in a randomised controlled trial of sixty-three participants for smoking reduction, found a reduction in nicotine dependence ratings, but no difference in serum cotinine and self-report consumption^(^ ^28^ ^)^. We could identify no prior publications exploring any relationships between maternal fish or *n*-3 consumption and smoking behaviours, before, during or after pregnancy.

In this study, we examined three questions regarding potential relationships between consumption of fish, as a rich source of *n*-3 HUFA, and perinatal smoking. We used data collected with standardised and validated protocols on 9640 mothers from the ALSPAC longitudinal cohort study: (1) was greater fish consumption associated with lower risks of smoking, at pre-conception and in each trimester?, (2) was greater fish consumption associated with greater likelihood of cessation of smoking, after conception, during each trimester?, and (3) was greater fish consumption associated with lower risks of smoking relapse between second and third trimesters, after pre-partum cessation? We sought to identify and adjust for confounding variables such as socioeconomic status that could underlie associations between greater fish consumption and healthier smoking behaviours.

## Methods

### Study population

The ALSPAC is a UK population-based study which aims to investigate environmental and genetic influences on the health and development of children^(^ ^29^ ^,^ ^30^ ^)^. Pregnant women residing in the former Avon Health Authority in South-West England who had an estimated date of delivery between 1 April 1991 and 31 December 1992 were invited to take part, resulting in a cohort of 14 541 pregnancies and 13 978 children alive at 12 months of age (excluding triplets and quads). Ethical approval for this study was obtained from the ALSPAC Law and Ethics Committee and the Local Research Ethics Committees. The representative nature of the ALSPAC sample has been investigated by comparison with the 1991 National Census data of mothers with infants under 1 year of age who were residents in the county of Avon. The ALSPAC sample had a slightly greater proportion of mothers who were married or cohabiting, who were owner-occupiers and who had a car in the household. The study had a smaller proportion of ethnic minority mothers. The ALSPAC study website contains details of all the data that are available through a fully searchable data dictionary (www.bris.ac.uk/alspac/researchers/data-access/data-dictionary/).

### Reported intake of fish and *n*-3 fatty acids

ALSPAC participant mothers completed a self-administered semi-quantitative FFQ during the third trimester of their pregnancy; median gestational age at completion was 32 weeks. As fish is the richest source of HUFA, we utilised three questions from the FFQ relating to fish consumption: How many times nowadays do you eat (a) white fish (cod, haddock, plaice, fish fingers, etc.), (b) dark or oily fish (tuna, sardines, pilchards, mackerel, herring, kippers, trout, salmon, etc.), and (c) shellfish (prawns, crabs, cockles, mussels etc.)? as previously described^(^ ^31^ ^)^. Responses to all food questions were used to derive a measure of total energy intake (in kJ (kcal)).

#### Fish consumption by weight

Portion sizes were based on typical eating patterns in the UK with consideration of relative proportions of processed and fresh fish commonly consumed^(^ ^31^ ^)^. Total fish consumption per week was calculated as the total number of portions, multiplied by the portion size for each type of fish. A mother who ate fish three times a week would typically have a fish intake of 347 g/week (range 297–358 g/week). This continuous measure which was guided by the US FDA/EPA advice to limit fish consumption to 340 g/week and was collapsed into a three-category ordinal measure comprising ‘no fish consumption’ (coded 0), ‘low fish consumption (1–340 g/week)’ (coded 1) ‘and high fish consumption (>340 g/week)’ (coded 2)^(^ ^31^ ^)^.

#### *n*-3 Intake

Fatty acid values were based on profiles of typical species of British fish^(^ ^32^ ^)^. Intake of *n*-3 fatty acids for each portion were estimated as follows: white fish, 0·32 g; oily fish, 0·89 g; shellfish, 0·34 g. Derived estimates of the intake of *n*-3 fats from fish: *α*-linolenic acid, EPA, DPA and DHA were calculated as total amounts and as proportions of total energy intake. This continuous measure was divided into six categories comprised of those with zero exposure (no fish consumption – coded 0) and five equally sized groups describing increasing exposure (coded 1 through 5). The exact cut-points on the distribution were selected attempting to maintain an adequate sample within each category and reflect clusters in the frequencies of responses.

### Measures of smoking behaviour

Information on tobacco use before and during pregnancy was collected via a postal questionnaire administered during mid pregnancy with further subsequent questionnaires assessing late-pregnancy and postnatal use. The mid pregnancy questionnaire (median gestation at completion=19 weeks gestation, interquartile range (IQR)=18–21 weeks) provided three measures of tobacco use – smoking pre-pregnancy, smoking in first trimester and smoking in second trimester. For each measure, responses on an ordinal scale determining frequency of use (cigarettes per d) were collapsed into a binary yes/no measure. An indicator of smoking in the third trimester was derived from a further question included in the late-pregnancy questionnaire which also contained the FFQ mentioned above.

### Confounders

Multiple measures described below were considered as potential confounders for the diet-smoking relationship. These comprised established risk factors for smoking behaviour for which we felt the assumption of a causal predictive relationship with fish consumption could be justified. This approach led the exclusion of certain variables – including maternal mental health and partner smoking behaviour – which did not fulfil this specific requirement despite being anticipated to be associated with both exposure and outcome in our models. Confounders were included irrespective of their observed impact in the empirical models.

#### Socio-demographic measures

Data collected by questionnaire during the antenatal period comprised: housing tenure (coded as owned/mortgaged, privately rented, subsidised housing rented from council/housing association), Maternal educational attainment (coded as no high school qualifications, high school, beyond high school) and parity (coded as whether study child is 1st/2nd/3rd child or greater: family size being another proxy for socioeconomic status), Home overcrowding at enrolment (1+ persons per room), Maternal age at delivery of study child (<20 years, 20–24, 25–29, 30–34, 35–39, 40+ years), financial difficulties in pregnancy (scoring in the top centile of a scale derived from ordinal responses to questions assessing the degree to which the family have difficulties affording food, clothing, heating, rent or mortgage, and items needed for the baby) and parental social class (the highest social class of either parent) at enrolment based on the Registrar General’s classification of occupations: I (professional), II (managerial and technical), IIINM (skilled non-manual), IIIM (skilled manual) or lower IV/V (semiskilled and unskilled) and ethnicity of young person (white/non-white).

#### Indicators of healthy lifestyle

Whilst difficult to measure accurately, health-consciousness is likely to strongly influence both diet and also smoking behaviour. We selected the following which were felt to tap into this concept. First, the 18-week questionnaire which asked: ‘Compared with other pregnant women of your age, would you consider yourself to be: much more active/somewhat more active/about the same/somewhat less active/much less active’. In addition, the 32-week questionnaire which is also part of the FFQ asks: frequency of consuming herbal teas (often/occasionally/never), and whether mother often eats/drinks other health foods.

### Statistical methods

A series of univariable and multivariable regression models were estimated using first fish consumption (three-category ordinal) and second *n*-3 from fish (six category ordinal) as the exposure variable. Regression estimates were derived with reference to the low fish consumption and *n*-3 HUFA-free category respectively. The binomial family and log-link were employed to derive risk-ratios given the relatively high number of cases (e.g. rate of smoking) for some models which would have detrimentally impacted on the interpretation of estimated OR. Models were subsequently adjusted for the potential confounding effects of socio-demographics and additionally health-lifestyle indicators. A consideration of the use of negative controls in this analysis is included in the supplementary materials. More details regarding the analytical approach used to address each aim are given below.

#### Aim 1: association between *n*-3/fish consumption and smoking status

A series of regression models were derived predicting smoking status at four different time points (i.e. pre-pregnancy, early pregnancy, mid pregnancy, late pregnancy). The sample used for this aim comprised all mothers with available data.

#### Aim 2: association between *n*-3/fish consumption and smoking cessation

A series of regression models were derived predicting smoking cessation at three different time points (i.e. early pregnancy, mid pregnancy, late pregnancy). The sample used for this aim comprised all mothers who reported smoking regularly before the pregnancy.

#### Aim 3: association between *n*-3/fish consumption and smoking relapse from a state of cessation

A final regression model was derived predicting smoking relapse in the third trimester of pregnancy. The sample used for this aim comprised all mothers who reported smoking regularly before the pregnancy and subsequent cessation by the second trimester.

**Fig. 1:**
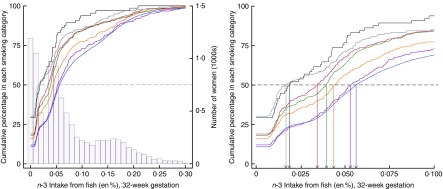
Cumulative percentage of smoking in pregnancy by *n*-3 fatty acid consumption. The relationships between levels of *n*-3 consumption and the cumulative percentage of women in each smoking category at 32 weeks gestation are illustrated. 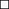, Number of women at each level of *n*-3 fatty acid intake from fish; coloured lines identify each smoking category defined by amount of cigarette consumption. The increasing cumulative percentages of women within each smoking category are indicated as a response to increasing *n*-3 intake. 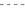, a cumulative threshold of 50 % of women detected within each smoking category. The intersection of the dashed line with each of the coloured lines, indicates the *n*-3 value for the median split of women in each smoking category. For example, a consumption level of 0·01 energy percentage (en%) *n*-3 accounts for 50 % of women in the smoking category of 20–24+ cigarettes/d. In contrast, a consumption level of 0·06 en% level of *n*-3 accounts for 50 % of women in the smoking category of 0 cigarettes/d. The right-hand panel is an expanded view identifies the specific levels of *n*-3 intake where the 50 % threshold is reached within each smoking category and indicates that higher levels of *n*-3 intake are associated with less smoking in pregnancy in a dose–response relationship. Cigarette consumption: 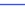, 0/d (*n* 7735); 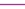, 1–4/d (*n* 300); 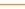, 5–9/d (*n* 458); 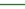, 10–14/d (*n* 535); 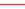, 15–19/d (*n* 317); 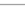, 20–24/d (*n* 230); 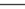, 25+/d (*n* 65).

## Results

### Sample derivation

The ALSPAC study enrolled mothers-to-be using a range of recruitment methods including posters displayed in chemists, libraries, general practitioner waiting rooms and antenatal clinics; coverage in the press, on radio and television, and through contact with midwives. Consequently gestation at enrolment varied markedly depending on each mother’s use of these services as well as her own awareness regarding her pregnancy status. Four questionnaires were administered to mothers during pregnancy and the timing and ordering of these questionnaires was determined by gestation at enrolment as set out in the study protocol. For mothers enrolling late in pregnancy, a fewer number of questionnaires were administered with the more time-sensitive questions removed. The majority of the analyses in this manuscript required responses from both the 18-week and 32-week gestation questionnaires. Of a possible available sample of 13 798, data were available from 10 519 (76·2 %) when excluding those who either: (i) failed to respond to one of the two questionnaires (*n* 1865), or (ii) responded to one or both questionnaires outside of the intended time window (including after the birth) such that their responses could be deemed as no longer representative of the pregnancy periods of interest (*n* 1414). The online Supplementary Table S1 shows that certain groups, for example, younger mothers and those from subsidised housing were under-represented in this sub-sample of 10 519.

Of the sample of 10 519 who returned both questionnaires at the appropriate time, a total of 9640 (91·6 %) respondents provided information on both antenatal smoking (18 and 32 weeks) and diet, 63 (0·60 %) lack information on diet alone, 804 (7·6 %) lack information on smoking alone (mainly from the 32-week questionnaire), and 12 (0·11 %) lack information both on diet and smoking. Of concern here was the considerable number failing to provide information on their third-trimester smoking behaviour, however there was little evidence of a difference in *n*-3 intake from diet for these mothers compared with members of the sample of 9640 (*t* test: *P*=0·2).

### Aim 1: risk of smoking before and during pregnancy (Table 1; Fig. 1)

Focusing on the sample of 9640 for which univariable complete-case analyses were possible, a total of 1194 (12·4 %) mothers report no fish consumption, the majority (6254, 64·9 %) consumed some fish but <340 g/week and 2192 (22·7 %) were estimated to consume 340 g or more per week.

*n*-3 Intake via fish consumption was estimated to account for between 0 and 0·74 % of total energy intake whilst energy intake per d ranged from 2178·2 to 16 954 kJ (520·6 to 4052 kcal). For those reporting no fish consumption, the distribution of energy intake had a median of 6586 kJ (IQR 5385–7904) (1574 kcal (IQR 1287–1889)). For those reporting <340 g of fish per week, the distribution of *n*-3 intake had a median of 0·046 % (IQR 0·025 %–0·070 %) and the distribution of energy intake had a median of 7138 kJ (IQR 5899–8431) (1706 kcal (IQR 1410–2015)). For those reporting fish consumption equivalent to 340 g or more per week, the distribution of *n*-3 intake had a median of 0·17 % (IQR 0·13–0·21) and the distribution of energy intake had a median of 7791 kJ (IQR 6611–9104) (1862 kcal (IQR 1580–2176)).

Categorisation of continuous *n*-3 measure from fish in diet (en%) was as follows: one group comprised of the 1194 (12·4 %) reporting no fish consumption and five equal groups of 17·5 % of the sample captured increasing levels of exposure. Five equally sized groups of those exposed to *n*-3 through fish in diet correspond to the following range of values: group 1 (median=0·013 %, range=0·006–0·030), group 2 (median=0·042 %, range=0·030–0·050), group 3 (median=0·061 %, range=0·050–0·078), group 4 (median=0·110 %, range=0·078–0·150), group 5 (median=0·190 %, range=0·150–0·740).

#### Fish consumption

In all, 3044 (31·6 %) reported smoking before the pregnancy. This reduced to 2260 (23·4 %) in the first trimester, 1789 (18·6 %) in the second trimester with a slight rise to 1905 (19·8 %) in the third trimester. The estimated association between fish consumption and risk of smoking is shown in Table 1. Before adjustment for confounders, there was strong evidence (*P*<0·001) of a large negative association between fish consumption and smoking. Lower fish consumption (<340 g/week) was associated with a 25 % lower risk of smoking, whilst high fish consumption (340 g+/week) was associated with a 50 % lower risk. Whilst the prevalence of smoking in the population diminished, from a high of 31·6 % (pre-pregnancy) to a low of 18·7 % (second trimester), the magnitude of effects for fish consumption were stable. Adjustment for confounders led to a substantial attenuation in the effect estimates, however there remained fairly strong evidence (*P* values in range 0·011–0·001) of a moderate beneficial effect of fish consumption, particularly for those who consumed 340 g or more per week where risk of smoking is approximately 20 % lower. Further investigation revealed that the confounders producing the greatest parameter attenuation were socio-demographics – maternal education, maternal age, social class and housing tenure.

**Table 1:** Aim 1, *n*-3 exposure/fish consumption and smoking (complete-case analyses) (Numbers and percentages; odds ratios and 95 % confidence intervals)

		Smoking		Unadj-RR (*n* 9640)		Adj-RR1 (*n* 8747)		Adj-RR2 (*n* 8102)	
Outcomes	Exposure	*n*	%	OR	95 % CI	OR	95 % CI	OR	95 % CI
Smoking pre-pregnancy	Fish consumption								
	None	505	42·3	1·00	Ref.	1·00	Ref.	1·00	Ref.
	<340 g/week	2021	32·3	0·76	0·71, 0·82	0·95	0·88, 1·02	0·94	0·87, 1·02
	340 g/week+	518	23·6	0·56	0·51, 0·62	0·84	0·76, 0·93	0·84	0·75, 0·94
				*P*<0·001		*P*=0·004		*P*=0·008	
	Linear effect of *n*-3 from fish (en%)			0·88	0·87, 0·90	0·96	0·95, 0·98	0·96	0·94, 0·98
				*P*<0·001		*P*<0·001		*P*<0·001	
Smoking in 1st trimester	Fish consumption								
	None	396	33·2	1·00	Ref.	1·00	Ref.	1·00	Ref.
	<340 g/week	1510	24·1	0·73	0·66, 0·66	0·92	0·83, 1·01	0·92	0·83, 1·02
	340 g/week+	354	16·2	0·49	0·49, 0·55	0·78	0·68, 0·89	0·80	0·69, 0·92
				*P*<0·001		*P*=0·002		*P*=0·011	
	Linear effect of *n*-3 from fish (en%)			0·85	0·83, 0·87	0·94	0·92, 0·97	0·95	0·92, 0·97
				*P*<0·001		*P*<0·001		*P*<0·001	
Smoking in 2nd trimester	Fish consumption								
	None	324	27·1	1·00	Ref.	1·00	Ref.	1·00	Ref.
	<340 g/week	1188	19·0	0·70	0·63, 0·78	0·87	0·77, 0·99	0·89	0·78, 1·01
	340 g/week+	277	12·6	0·47	0·40, 0·54	0·72	0·61, 0·85	0·75	0·63, 0·90
				*P*<0·001		*P*<0·001		*P*=0·009	
	Linear effect of *n-*3 from fish (en%)			0·84	0·82, 0·87	0·93	0·90, 0·96	0·94	0·91, 0·97
				*P*<0·001		*P*<0·001		*P*<0·001	
Smoking in 3rd trimester	Fish consumption								
	None	353	29·6	1·00	Ref.	1·00	Ref.	1·00	Ref.
	<340 g/week	1261	20·2	0·68	0·62, 0·75	0·88	0·79, 0·99	0·87	0·77, 0·97
	340 g/week+	291	13·3	0·45	0·39, 0·52	0·73	0·62, 0·85	0·73	0·61, 0·86
				*P*<0·001		*P*<0·001		*P*=0·001	
	Linear effect of *n*-3 from fish (en%)			0·84	0·82, 0·86	0·93	0·90, 0·96	0·93	0·91, 0·96
				*P*<0·001		*P*<0·001		*P*<0·001	

RR, relative risk; Adj-RR1, adjustment for socio-demographics, that is maternal education, maternal age at delivery, housing tenure, home overcrowding, birth order, parental highest social class, financial problems in pregnancy and child ethnicity; Adj-RR2, further adjustment made for indicators of a healthy lifestyle, that is perception of activity levels in early pregnancy compared with peers, consumption of herbal tea and health foods in late pregnancy; Ref., referent values; en%, energy percentage.

#### *n*-3 Highly unsaturated fatty acids

There was good support for a linear relationship (dose–response) between categories of estimated *n*-3 intake from fish (as % energy) and risk of smoking (further details are available in the online Supplementary Appendix). Table 1 shows the estimated linear effect, namely the reduction in risk per category increase in *n*-3. Results mirror those for fish consumption with a moderate benefit in terms of reduced smoking risk for each category increase in *n*-3 exposure.

### Aim 2: likelihood of smoking cessation inpregnancy (Table 2)

The likelihood of smoking cessation among those mothers previously reporting tobacco use was examined. The starting sample for these analyses was the 3044 (31·6 %) reporting smoking regularly before the pregnancy. As expected, the rates of fish consumption among this subgroup were slightly lower: a total of 505 (16·6 %) reported no fish consumption, 2021 (66·4 %) consumed some fish, but <340 g/week and 518 (17·0 %) consumed 340 g or more per week.

#### Fish consumption

Cessation rates increased between trimesters 1 and 2 (from 26·1 % to 41·5 %), before reducing slightly to 38·6 % in trimester 3 as some mothers relapse again. There was strong evidence (*P*≤0·002) for a moderate positive association between fish consumption and smoking cessation amongst this subgroup of recent regular smokers (Table 2). Adjustment for potential confounders once again leads to substantial attenuation.

**Table 2:** Aim 2 – *n*-3 exposure/fish consumption and smoking cessation (complete-case analyses) (Numbers and percentages; odds ratios and 95 % confidence intervals)

		Not smoking		Unadj-RR (*n* 3044)		Adj-RR1 (*n* 2615)		Adj-RR2 (*n* 2402)	
Outcome	*n*-3 Exposure	*n*	%	OR	95 % CI	OR	95 % CI	OR	95 % CI
Cessation in 1st trimester	Fish consumption								
	None	111	22·0	1·00	Ref.	1·00	Ref.	1·00	Ref.
	<340 g/week	517	25·6	1·16	0·97, 1·39	1·02	0·84, 1·24	1·02	0·83, 1·25
	340 g/week+	166	32·0	1·46	1·19, 1·79	1·23	0·98, 1·53	1·16	0·92, 1·47
				*P*<0·001		*P*=0·042		*P*=0·214	
	Linear effect of *n*-3 from fish (en%)			1·10	1·06, 1·14	1·06	1·02, 1·11	1·04	1·00, 1·08
				*P*<0·001		*P*=0·004		*P*=0·082	
Cessation in 2nd trimester	Fish consumption								
	None	183	36·2	1·00	Ref.	1·00	Ref.	1·00	Ref.
	<340 g/week	838	41·5	1·14	1·01, 1·30	1·07	0·94, 1·23	1·07	0·93, 1·23
	340 g/week+	243	46·9	1·29	1·12, 1·50	1·17	1·00, 1·37	1·12	0·95, 1·31
				*P*=0·002		*P*=0·109		*P*=0·384	
	Linear effect of *n*-3 from fish (en%)			1·06	1·04, 1·09	1·04	1·01, 1·07	1·02	0·99, 1·05
				*P*<0·001		*P*=0·007		*P*=0·110	
Cessation in 3rd trimester	Fish consumption								
	None	162	32·1	1·00	Ref.	1·00	Ref.	1·00	Ref.
	<340 g/week	777	38·5	1·20	1·04, 1·38	1·06	0·92, 1·23	1·09	0·95, 1·27
	340 g/week+	235	45·6	1·41	1·21, 1·66	1·18	1·01, 1·39	1·13	0·96, 1·34
				*P*<0·001		*P*=0·076		*P*=0·341	
	Linear effect of *n*-3 from fish (en%)			1·08	1·06, 1·11	1·04	1·01, 1·07	1·03	1·00, 1·06
				*P*<0·001		*P*=0·003		*P*=0·086	

RR, relative risk; Adj-RR1, adjustment for socio-demographics, that is maternal education, maternal age at delivery, housing tenure, home over-crowding, birth order, parental highest social class, financial problems in pregnancy and child ethnicity; Adj-RR2, further adjustment made for indicators of a healthy lifestyle, that is perception of activity levels in early pregnancy compared with peers, consumption of herbal tea and health foods in late pregnancy; Ref., referent values; en%, energy percentage.

#### *n*-3

An identical pattern was found here: (i) the change in risk of cessation is essentially monotonic across categories of *n*-3 (online Supplementary Appendix), (ii) the results mirrored those for fish consumption with lower *P* values here due to increased power.

### Aim 3: risk of smoking relapse in mid pregnancy (Table 3)

Aim 3 focused on a relapse in smoking between the second and third trimester, that is, members of the sample reported smoking regularly before pregnancy and were not smoking in the second trimester (*n* 1264). A total of 237 were smoking when asked subsequently at 32 weeks gestation giving a relapse rate of 18·8 %. Fish consumption in this subgroup was slightly higher than that for Aim 2: 183 (14·5 %) reported no fish consumption, 838 (66·3 %) consumed some fish but <340 g per week and 243 (19·2 %) consumed 340 g or more per week (Table 3).

**Table 3:** Aim 3 – relapse model, *n*-3/fish consumption and relapse between second and third trimester (complete-case analyses) (Numbers and percentages; odds ratios and 95 % confidence intervals)

	Relapsed		Unadj-RR (*n* 1264)		Adj-RR1 (*n* 1125)		Adj-RR2 (*n* 1024)	
Exposures	*n*	%	OR	95 % CI	OR	95 % CI	OR	95 % CI
Fish consumption								
None	46	25·1	1·00	Ref.	1·00	Ref.	1·00	Ref.
<340 g/week	155	18·5	0·74	0·55, 0·98	0·98	0·57, 1·66	0·99	0·56, 1·73
340 g/week+	36	14·8	0·59	0·40, 0·87	0·89	0·47, 1·68	0·92	0·39, 2·19
			*P*=0·023		*P*=0·903		*P*=0·966	
Linear effect of *n*-3 from fish (en%)			0·89	0·84, 0·96	0·97	0·87, 1·09	0·99	0·85, 1·16
			*P*=0·001		*P*=0·607		*P*=0·919	

RR, relative risk; Adj-RR1, adjustment for socio-demographics, that is maternal education, maternal age at delivery, housing tenure, home over-crowding, birth order, parental highest social class, financial problems in pregnancy and child ethnicity; Adj-RR2, further adjustment made for indicators of a healthy lifestyle, that is perception of activity levels in early pregnancy compared with peers, consumption of herbal tea and health foods in late pregnancy; Ref., referent values; en%, energy percentage.

Whilst fish consumption and in particular >340 g/week was protective against smoking relapse in the unadjusted analyses, the results attenuated substantially following adjustment for confounders leaving little evidence of any beneficial effect in the multivariable models.

## Discussion

### Summary of findings

Here we found that among pregnant women in the UK, greater fish consumption had a modest, but consistent association with lower risk of smoking in the perinatal period and in all three trimesters of pregnancy. These associations had a clear dose–response relationship concordant with modelling protective levels of *n*-3 HUFA intake , with a 4–7 % reduction in prevalence for each increasing quintile of *n*-3 HUFA intake, relative to mothers who reported never eating fish. Consumption of greater than 340 g fish per week was consistently associated with beneficial smoking behaviours. These finding persisted after controlling for both socio-demographic factors and indicators of healthy life-style practices.

The prevalence of smoking decreased as weeks of gestation increased from approximately 32 % pre-pregnancy as to a low of 19 % during the second trimester. However, despite this reduction in smoking rates across trimester, the magnitude of protective effects for fish consumption were consistent. Protective relationships, similar in magnitude and direction, were observed for fish consumption and smoking cessation across trimesters. However, although these relationships were robust to adjustment for confounding by socio-demographics factors, they were not robust to further adjustment by factors specifically indicative of a healthy lifestyles. Thus, fish consumption may have been an indicator of greater overall health conscious behaviours, including less smoking.

Given the well-established risks of harm from maternal smoking including missing or deformed limbs, cleft palate, premature birth and cot death^(^ ^2^ ^)^, the possible identification of any intervention with a safe an beneficial profile with potential to reduce smoking during pregnancy, is useful. Rabinovitz *et al.* ^(^ ^25^ ^)^ recently reported a significant decrease in self-reported daily smoking and tobacco cravings in regular cigarette smokers (*n* 48) consuming 2710 mg of EPA and 2040 mg of DHA daily for one month in the context of a randomised, placebo-controlled pilot study^(^ ^25^ ^)^. Currently, the main pharmacological treatments for smoking cessation include bupropion (dopamine reuptake inhibitor), varenicline (partial agonist of nicotine receptors), nicotine replacement therapy and electronic cigarettes (non-prescribed). All present with side-effects including disrupted sleep, headaches, nausea, tachycardia and dry mouth^(^ ^26^ ^)^. The effectiveness of these therapies for pregnant mothers has been reported as limited in many systematic reviews. In 2012 Cochrane report reviewed thirty-two pharmacologic agents for smoking cessation but found insufficient evidence to permit conclusions about benefits and harms^(^ ^33^ ^)^. In contrast, the safety profile of fish an *n*-3 fatty acids in pregnancy have been well described. The Food Drug Agency (FDA) currently advises that that dietary dosage of up to 3 g/d of *n*-3 fatty acids from marine sources are ‘generally recognised as safe’. While the European Food Standards Authority have set a safe upper limit of 5 g/person per d for total amounts of the *n*-3 fatty acids: DHA, EPA and DPA.

### Potential biological mechanisms

The mechanisms of how *n*-3 HUFA and nutrients rich in fish might impact the neurobiology underlying tobacco use and addictive processes are complex; more than eighteen neurotransmitter systems across multiple neurotransmitter systems and brain regions involving multiple stages of development of drug-seeking habits and maintenance^(^ ^34^ ^)^. Dietary deficits in *n*-3 HUFA appear to contribute to many of these central characteristics of the chronic addicted state including; deficits in dopaminergic components of the reward system^(^ ^35^ ^–^ ^38^ ^)^, hyperactivity of the endocannabinoid system^(^ ^39^ ^,^ ^40^ ^)^, impaired neurogenesis^(^ ^41^ ^,^ ^42^ ^)^ and excessive recruitment of brain stress neurotransmitters, including corticotropin-releasing factor^(^ ^43^ ^,^ ^44^ ^)^ expressed in the neurocircuitry of the extended amygdala. DHA is enriched throughout neuronal members and especially concentrated among cortical grey matter and in brain regions including the corpus callosum, frontal, temporal and parietal lobes, and amygdala^(^ ^45^ ^,^ ^46^ ^)^. Most of the accumulation of DHA takes place during prenatal and early postnatal development^(^ ^41^ ^,^ ^42^ ^,^ ^45^ ^,^ ^47^ ^)^ and *n*-3 HUFA are implicated in neurogenesis, synaptogenesis, and myelination^(^ ^41^ ^,^ ^42^ ^)^.

Both DHA and EPA have modulating effects on dopaminergic and serotonergic systems and in animal models chronic *n*-3 HUFA deficiency results in marked alterations in dopamine-related neurotransmission and behaviour^(^ ^35^ ^–^ ^38^ ^)^. Low *n*-3 impacts dopamine neurotransmission resulting in hypofunctioning of the mesocorticial systems associated with reward and dependence^(^ ^35^ ^)^. Conversely, restoration and elevation of *n*-3 is able to reverse deficits in dopaminergic and serotonergic neurotransmitter function^(^ ^36^ ^)^. This diminished function of the mescocortical systems may contribute to increased craving responses and thus negatively interfere with smoking cessation attempts^(^ ^25^ ^,^ ^26^ ^)^. Deficits in dopaminergic, serotinergic neurotransmission and hyper-responsive stress reactivity mediated by corticotrophin releasing factor are associated with increased risks of negative affective emotional states that mediate increased risk of relapse to substance use^(^ ^48^ ^–^ ^51^ ^)^.

#### Strengths and limitations of study

The major strength of the study is the large sample size and the careful controlling of confounding variables. There are some limitations of note, namely, limits inherent in food frequency questionnaires, the potential of misclassification in measurements and the self-report smoking records. We recognise also that the chronological order of data collection is imperfect. Specifically, data on the predictive variable, fish consumption, was collected at 32 weeks, whereas data on dependent variable of smoking behaviours were collected before this time point. However, as dietary habits are relatively consistent across time so too fish consumption is likely consistent. We wish to emphasise that residual confounding and reverse causality cannot be ruled out. Thus, we cannot establish with certainty, any causal relationships demonstrating efficacy between fish or *n*-3 consumption and more beneficial smoking behaviours.

### Conclusion

Here we found modest but consistent evidence of a beneficial association between fish consumption during pregnancy and more beneficial smoking behaviours during pregnancy, after evaluation and adjustment for confounding variables. Lower risks of adverse smoking behaviours were found among mothers consuming 340 g or more per week. We suspect that *n*-3 HUFA may substantially contribute to this association these agents, or greater fish consumption can be considered for evaluation in secondary and tertiary preventive intervention trials for smoking in pregnant women. A recent study reporting lower *n*-3 HUFA among non-pregnant smokers is consistent with this interpretation^(^ ^52^ ^)^. These findings may provide a basis of support for the conduct of randomised controlled trials designed to evaluate if greater fish consumption, or nutrients rich in fish such as *n*-3 fatty acids, has clinical efficacy in improving smoking cessation or in reducing risk of smoking.

## Supplementary material

For supplementary material accompanying this paper visit http://dx.doi.org/10.1017/S0007114517003592. click here to view supplementary material
