# Supplementary material for: Maternal fish consumption during pregnancy and smoking behavioural patterns
Source: Br J Nutr. 2018 Mar 28;119(11):1303–11. doi: 10.1017/S0007114517003592 (PMC6088548; doi:10.1017/S0007114517003592)
Supplement: Supplementary file 1 [file S0007114517003592sup001.docx]

**Assessment of negative controls**

Negative control exposures or outcomes are used to address a number of concerns regarding one’s analysis – such as selection bias or measurement error – however here we felt the potential for residual confounding was of concern. In particular, as mentioned in the manuscript, we felt that health-conscious behaviour was both important and difficult to measure hence this is a prime candidate for introducing residual confounding.

Fish

Smoking

Measured confounders

Health-conscious living
(unmeasured confounder)

Other healthy foods

Our assumed DAG for this analysis is shown above. The association between fish consumption and smoking behaviour may be confounded by health-conscious behaviour which is unmeasured or poorly measured in this setting. We theorize that fish consumption is (causally) related to smoking behaviour due to fish being a rich source of HUFAs. If this were the case the other healthy foods which did not contain HUFAs would be related to fish consumption due to the measured and unmeasured confounders but would not be causally related to smoking. Other healthy foods could then be regarded as a Negative Control (NC). The same argument could be made for unhealthy foods, therefore from the same FFQ which gave us the measure of fish consumption we selected individual measures assessing consumption of salad, pulses and fresh fruit (healthy) and chips, fried food and sweets (unhealthy). Here “fried foods” referred specifically to common ingredients of a cooked “English” breakfast rather than for example the type of fish that might accompany chips.

We follow the approach discussed in Flanders et al (2011) which states that in a sequence of models determining the effect of both the exposure (fish) and (separately) the NC on an outcome when adjusting for measured confounders, when subsequently mutually adjusting the exposure and the NC one should see attenuation of the effect for the NC but little other than a change in precision for the exposure. To reduce the number of parameter estimates, and due to the dose-response effect reported for fish in the manuscript, we consider all food-types as linear predictors in these analyses. We focus on the measure of smoking behaviour at 32 weeks gestation. Results are shown in Table 1.

*Interpretation*

The estimates shown in Table 1 show that contrary to the situation described by Flanders et al (2011) there is no evidence that the effect estimate for the NC attenuates to any greater or lesser extent than that for fish consumption, although in both instances the reduction in the effects are relatively minor compared to that seen on adjustment for socio-demographic measures.

In our view to make sense of these findings we must recognise the fact that the use of negative controls is far less robust outside of the laboratory where things can be manipulated experimentally. Consequently, we propose that whilst a null finding for these NC’s would support our assertion, the lack of one does not actually refute them.

We refer to Uddin et al (2016) who comments:

*“ …a non-null association will only suggest unmeasured confounding for the relation between the NC exposure and the outcome (or exposure and NC outcome) and does not necessarily indicate unmeasured confounding of the exposure–outcome relation of interest”*

*Summary*

In conclusion, we sought to identify negative controls to evaluate the possibility that health conscious behaviour was a substantial driver of the relationship between fish consumption and smoking behaviours. We selected items from the food frequency questionnaire that were strongly indicative of either having, or alternatively not, having health conscious behaviours. The inclusion of the variable measuring fish consumption did not substantially attenuate these positive and negative relationships to smoking behaviours. However, our failure to find attenuations of relationship does not necessarily mean that no such relationship exist. Therefore, we cannot conclude with reasonable certainty that there is, or is not, evidence of residual confounding due to health-conscious behaviour based on the negative controls employed. If the editors/reviewers have any specific suggestions as to how to operationalize this negative control examination with other parameters, we welcome these suggestions.

**References**

Flanders WD, Klein M, Darrow LA, Strickland MJ, Sarnat SE, Sarnat JA, et al. A method for detection of residual confounding in time-series and other observational studies. *Epidemiology*. 2011;22(1):59–67.

Uddin, M. J., Groenwold, R. H. H., Ali, M. S., de Boer, A., Roes, K. C. B., Chowdhury, M. A. B., & Klungel, O. H. (2016). Methods to control for unmeasured confounding in pharmacoepidemiology: an overview. *International Journal of Clinical Pharmacy*, 38, 714-723.

Table 1. Results of Negative Control analysis

|  |  | Effect of NC  in each model | | | | Effect of fish consumption  in each model | | | |
| --- | --- | --- | --- | --- | --- | --- | --- | --- | --- |
| NC-included |  | RR | Lower CI | Upper CI | p-value | RR | Lower CI | Upper CI | p-value |
| None | UOR |  |  |  |  | 0.674 | 0.629 | 0.722 | <0.001 |
|  | Adj1 |  |  |  |  | 0.859 | 0.796 | 0.927 | <0.001 |
|  | Adj2 |  |  |  |  | 0.857 | 0.789 | 0.930 | <0.001 |
| chips | UOR | 0.653 | 0.614 | 0.695 | <0.001 |  |  |  |  |
|  | Adj1 | 0.895 | 0.842 | 0.952 | <0.001 |  |  |  |  |
|  | Adj2 | 0.917 | 0.859 | 0.979 | 0.010 |  |  |  |  |
|  | Adj3 | 0.915 | 0.858 | 0.977 | 0.008 | 0.854 | 0.786 | 0.928 | <0.001 |
| Fried food | UOR | 0.693 | 0.662 | 0.724 | <0.001 |  |  |  |  |
|  | Adj1 | 0.835 | 0.795 | 0.876 | <0.001 |  |  |  |  |
|  | Adj2 | 0.836 | 0.794 | 0.879 | <0.001 |  |  |  |  |
|  | Adj3 | 0.839 | 0.797 | 0.882 | <0.001 | 0.862 | 0.794 | 0.936 | <0.001 |
| Salad | UOR | 0.762 | 0.733 | 0.793 | <0.001 |  |  |  |  |
|  | Adj1 | 0.898 | 0.859 | 0.939 | <0.001 |  |  |  |  |
|  | Adj2 | 0.908 | 0.867 | 0.950 | <0.001 |  |  |  |  |
|  | Adj3 | 0.918 | 0.876 | 0.961 | <0.001 | 0.872 | 0.803 | 0.948 | 0.001 |
| Fruit | UOR | 0.617 | 0.592 | 0.643 | <0.001 |  |  |  |  |
|  | Adj1 | 0.784 | 0.745 | 0.825 | <0.001 |  |  |  |  |
|  | Adj2 | 0.781 | 0.741 | 0.823 | <0.001 |  |  |  |  |
|  | Adj3 | 0.789 | 0.748 | 0.832 | <0.001 | 0.907 | 0.834 | 0.986 | 0.022 |
| Sweets | UOR | 0.848 | 0.815 | 0.883 | <0.001 |  |  |  |  |
|  | Adj1 | 0.943 | 0.900 | 0.988 | 0.015 |  |  |  |  |
|  | Adj2 | 0.952 | 0.905 | 1.000 | 0.050 |  |  |  |  |
|  | Adj3 | 0.951 | 0.905 | 0.999 | 0.045 | 0.856 | 0.788 | 0.929 | <0.001 |
| Pulses | UOR | 0.684 | 0.625 | 0.748 | <0.001 |  |  |  |  |
|  | Adj1 | 0.894 | 0.809 | 0.988 | 0.029 |  |  |  |  |
|  | Adj2 | 0.941 | 0.841 | 1.053 | 0.288 |  |  |  |  |
|  | Adj3 | 0.951 | 0.850 | 1.063 | 0.377 | 0.858 | 0.790 | 0.931 | <0.001 |

All estimates are risk-ratios given prevalence of smoking outcome. Unhealthy foods have been reverse coded so all estimates are for what would be expected to be an increasingly beneficial behaviour.

Adj1 = adjustment for socio-demographics, i.e. maternal education, maternal age at delivery, housing tenure, home over-crowding, birth order, parental highest social class, financial problems in pregnancy and child ethnicity.

Adj2 – further adjustment made for indicators of a healthy-lifestyle, i.e. perception of activity levels in early pregnancy compared to peers, consumption of herbal tea and health-foods in late pregnancy.

Adj3 – further adjusted for fish consumption
